# Perceived academic stress during a pandemic: Mediating role of coping strategies

**DOI:** 10.1016/j.heliyon.2023.e16594

**Published:** 2023-06-01

**Authors:** Habeeb Ur Rahiman, Niyaz Panakaje, Abhinandan Kulal, Harinakshi, S M Riha Parvin

**Affiliations:** a College of Business Administration, Kingdom University, Bahrain; b The Yenepoya Institute of Arts, Science, Commerce & Management, Yenepoya (Deemed to be University), Deralakatte, 575018, Mangalore, Karnataka, India; c Guest Faculty, Department of Commerce, University Evening College, Mangaluru, India; d Research Scholar, Institute of Management and Commerce, Srinivas University, Mangalore, India; e Institute of Management and Commerce, Srinivas University, Mangalore, India

**Keywords:** Academic stress, COVID-19, Coping strategy, Exam anxiety, Higher education

## Abstract

COVID-19 rampant has impacted almost all sections of society, and the repercussions were mostly negative experiences for people and have resulted by way of disruption in their daily routines. Academics is one such vital section that has suffered directly because of the inaccessibility of a comfortable educational procedure. Due to a shift in the form of education, most of the student community failed to obtain routine and regular education as the government entirely shuttered educational facilities to limit the spread of disease. In this light, this research attempted to examine the amount of academic stress experienced by students during the COVID-19 Pandemic and the strategies they have adopted to cope with this unheard type of uncertain situation. The findings of the study indicated substantial variations in Academic Stress, Exam Anxiety, and Coping Strategies across various demographic characteristics of the respondents. Another significant finding is that students from poor socio-economic backgrounds and those seeking post-graduate courses are more stressed. As an inference, it is also opined that to mitigate the impact of the COVID-19 crisis on student performance and psychological well-being, special focus, or techniques for accommodating exam environments by the student should be implemented. To minimize stress, the study also proposed efficient coping techniques to lower the amount of stress in various academic tasks.

## 1 Introduction

COVID-19, classified as a Public Health Emergency of International Concern by the World Health Organization (WHO) on January 30, 2020, and later reclassified as a pandemic on March 11, 2020, is currently considered one of the largest healthcare and economic disasters in history, impacting a number of countries around the globe [1]. The pandemic has had a profound impact on all aspects of human life, including the education sector. India, with 4.2 million verified COVID-19 cases and 71,642 deaths as of September 7, 2020, is the world's second-worst COVID-affected country, trailing only the United States of America [1]. In response to a spike in positive cases, the Indian government implemented a nationwide lockdown on March 25, 2020, to stop the spread of the disease. Academic institutions were compelled to temporarily close due to government directives, causing academic delivery to be hampered.

The COVID-19 pandemic has affected approximately 1.6 billion students in over 200 nations, resulting in significant changes in every part of academic life [2]. In India, academic institutions were closed due to government directives, and academic delivery was hampered during the lockdown period. The pandemic gave academic institutions an opportunity to prepare the road for digital learning to be introduced. However, transitioning from long-established face-to-face education to virtual education was a completely different scenario for both teachers and students. During the pandemic, e-learning platforms served a critical role in assisting schools and colleges in facilitating student education during the shutdown of schools and colleges. However, the teaching fraternity faced a significant issue in evaluating students' performance in an online class.

In the education system, exams are one type of tool used to evaluate a student's performance [3]. That has become a significant tool for evaluating a person's excellence or ability too [4] However, introducing a new system for the evaluation process and exposing the practice of teaching-learning virtually created stress among students. The epidemic has impacted students’ mental health all throughout the world. The most common difficulties among students studying for examinations are stress and worry [5] Stress is an unavoidable and beneficial component of academic life as it encourages students to work hard and stay on task. However, excessive stress can have a bad impact on one's health and well-being. It is essential to identify and differentiate between stress that helps students concentrate and stress that makes it difficult for them to study properly. Finding the factors that affect the academic performance of the students adversely can be eliminated by adopting some strategies.

Stress may be defined as any shift that causes physical, emotional, or psychological strain. It's a bodily reaction to anything that demands one’s attention or action [6] In India, fear, melancholy, numbness, sleeplessness, bewilderment, anger, post-traumatic stress symptoms, depression, low mood, tension, emotional dysregulation, and impatience were among the symptoms experienced by students during the lockdown period [7]. Exam delays have come from the COVID-19 outbreak, causing worry and anxiety among students [8]. Not only has the delay been annoying, but the uncertainty surrounding the administration of multiple competitive tests has harmed many candidates, perhaps influencing their outcomes [9,10]. The COVID-19 Pandemic had a significant influence on university students, and everyone has felt some amount of stress as a result of the closure of all institutions, widespread social isolation, and anxiety about the examination [11].

The COVID-19 pandemic has had a significant impact on the education system, with the closure of academic institutions and the need for remote learning. The transition to virtual education has been challenging for both teachers and students. The evaluation of students' performance has also been a challenge, with the introduction of new evaluation systems. The pandemic has had a significant impact on students' mental health, causing stress, anxiety, and other emotional and psychological difficulties. Strategies need to be adopted to eliminate factors that adversely affect academic performance, and stress management is critical. The delays and uncertainty surrounding the administration of exams due to the pandemic have caused additional stress and anxiety among students.

The COVID-19 pandemic has significantly impacted students' academic lives, leading to a range of issues, including exam anxiety, academic stress, and online learning difficulties. Several studies have shown that exam anxiety has a direct impact on academic stress, and coping strategies can help students cope with academic stress. However, there is limited research on the effectiveness of coping strategies during a pandemic and their potential impact on academic outcomes. Therefore, this study aims to investigate the relationship between perceived academic stress and coping strategies during a pandemic, with a specific focus on the mediating role of coping strategies in this relationship. The study also aims to identify the most common coping strategies used by students during a pandemic, explore potential interventions to improve coping strategies, and examine their impact on academic performance and motivation. The findings of this study could provide useful insights for educational institutions and policymakers to help students cope with academic stress during a pandemic.

### 1.1 Literature review

The abrupt closure of schools and colleges has disrupted the smooth flow of learning as well as the manner of delivering information and assessing students. Studies concerning university students comparing the periods before and during the COVID-19 pandemic revealed: in Portugal, higher levels of depression, anxiety, and stress during the pandemic period [12]; in Poland, a rise in the prevalence of anxiety as the pandemic continues to evolve [13]; in Italy, depression symptoms deteriorated during the lockdown, but by the end, they were equivalent to those documented before the COVID-19 outbreak [14] A research in the Brazilian state of So Paulo (Campinas municipality) found no significant difference in perceived stress and depressive symptoms [15]. A study that compared the period before and during the pandemic in the adult population of Brazil, discovered mild to serious anxiety and depression symptoms expanded by 6.6 & 7.4 times, respectively [16]. In this era, many institutions have taken the risk of conducting online tests; however, the effectiveness of online exams for evaluation and assessment is very speculative. Even if converting learning and teaching to virtual mode alleviated part of the stress, the education sector's largest issue was delivering exams in the middle of a health emergency. We cannot, however, infer that such institutions' actions were incorrect because universities that chose to postpone examinations for unknown dates caused ambiguity and worry among students, resulting in disproportionate academic stress when compared to ordinary exam settings. Hence, academic stress due to COVID-19 is a matter of concern [17]. Despite the fact that numerous studies have been conducted to quantify academic stress among students, they have failed to address the amount of academic stress in a pandemic environment [18]. Therefore, this research wanted to discover how online exam anxiety affects academic stress and what can be effective measures to reduce this stress among students. The researcher searched Google Scholar, Scopus Journals, and PubMed for existing literature using the keywords "exam anxiety" and "academic stress" in various permutations and combinations to locate the research gap and specify study variables. To identify the study variables, the researcher retrieved 56 papers and then classified them depending on their aims. In order to assess the impact of one variable on the other, the literature review has been categorized into four parts – a) Demographic factors b) Exam Anxiety and Academic Stress, c) Exam Anxiety and Coping Strategies, and d) Coping Strategies and Academic Stress which is further discussed below.

a) Demographic Factors

Previous studies for example [19] identified that the sudden changes took place in the education field during a pandemic, i.e., offline academic delivery to the online teaching-learning process, which was a burden for many families as some of the families lost their job due to COVID-19 and they were not in the position to buy electronic gadgets to their children. This was the hurdle that students have undergone stress at various levels. The study conducted [20] found that female students are emotionally down during COVID-19 and the same effected their studies. They were the ones who were involved in household chores and take care of their family members which made their focus deviate from studies. The shift of focus from studies to household chores started to make them anxious and stressed their academics. As a result of the lockdown and loss of work, most households lose their daily income, therefore inability to provide the necessities to their families. Levels of stress became significantly higher in students from low-income families during the COVID-19 period.

Zhang et al. [21], highlighted that inadequate network access and unawareness of online platforms like blackboard collaborate and zoom was discovered to be the main obstacles and interruptions experienced by the respondents. However, it was discovered that monetary concerns like declining income and rising expenses had minimal effect on online learning. According to Cannavò et al., [22], factors including gender, academic discipline, living situation, and history of quarantine also have an effect on how students view the online teaching-learning process. Despite countrywide attempts to create a solid infrastructure for e-learning, the majority of respondents in his study stated that they had never had such an experience [23]. Rogowska & Meres [24] viewed that students’ capacity for self-learning is increasingly important in remote learning. Most of the respondents stated that self-learning necessitates the student to maintain self-discipline, which can be challenging in the absence of direct teacher supervision. However, during the pandemic, it was challenging for students to get answers to many of their questions about the ideas covered in class. Furthermore, the study of Sharma & Mishra [25], found that the most important predictors of reported stress among students were age, educational level, students' lower levels of technology expertise, and students’ talents. Other studies also pondered that age, year of study, class, poor/low social support, economic status, career prospects, and region not only impacted academic stress but also influenced the adoption of coping strategies among medical students. All of these elements worked together to raise the stress level among students which directed researchers’ attention toward the influence of demographic factors on academic stress, online exam anxiety, and coping strategies. Based on the above discussion two hypotheses can be developed.

H_1_: There is a significant difference in online exam anxiety, Academic stress, and Coping strategy among demographic variables.

b) Exam Anxiety and Academic Stress:

Exam anxiety and academic stress are sometimes used interchangeably in many situations; however, research has revealed that they have different operational definitions. To put the two notions into context, stress is a physiological and behavioral response to an impending threat or challenge, whereas anxiety is an emotional and psychological condition produced by the dread of harm [26]**.** Exam stress and exam anxiety are theoretically separate as well. Exam anxiety is a sort of performance anxiety that happens when a person is formally or informally evaluated. It is most commonly experienced by children when taking tests, presentations, and engaging in class [27]**.** The concern of being adversely judged is a typical fear mentioned by test-anxious youngsters [28]**.** Academic stress, on the other hand, is described as the body's response to academic-related pressures that surpass students' adaptive capacity [29]. Because stress is defined in a broader context than anxiety, exams might be considered stressful by virtue of their inherent features or functions without referring to subjective fear and arousal [30]**.** Furthermore, it is anticipated that 10–30% of students will suffer some kind of academic stress during their academic careers [31]. As per the studies it has various dimensions and indicators consisting of academic stress caused due to exams, class meeting tasks, instructional methods, and lectures while working [32], a large amount of tasks, a large number of non-student peers, the duration of tutorials/lectures, and interacting with the opposite gender [33]**,** objective and subjective assessments, assignments and projects, attendance, pop quizzes, and class involvement [34], personal, interpersonal, and practical stress [35], academic course and coursework, course assessment methods, college instruction, campus social milieu, and cultural factors, college management and hierarchy, physical problems and accommodation facilities, economic forces, organismic and interactional factors, expectations, distress and constraints [36]. Also, academic stress is caused by the final and mid-term exams [37] as well as academic stress is caused by other people, personal causes, academic factors, and environmental variables [38].

After releasing the clear differences between the two concepts it is pertinent to understand their relationship. Further discussion on this context emphasizes that exam-related anxiety was found to be the most common source of anxiety among students in higher education [39]. It is the feeling of being nervous before and during a test and is very common among students [40]. As the current COVID-19 pandemic caused a dramatic change in online learning, traditional teaching-learning practices have been divested [41]. As a result, the choice to administer exams online increased which in turn uplifted the students’ anxiety [42] by causing them to worry about technological aspects [43]. Online exams have their own set of problems, such as a bad network, a lack of infrastructures like laptops and smartphones, and a lack of technical knowledge among teachers and students [44], unknown aspects of the online mode, a time limit for the exam, difficulties accessing the question paper, delays in uploading, technical issues, internet connectivity, and the fairness of the online mode, etc. These exam anxieties have caused academic stress among students [45] however, support from family and teachers helps to reduce academic stress. This Academic stress is nothing, but the emotional distress associated with academic tasks, disasters, or even knowing the possibility of academic failure [46]. The most common causes of this academic stress include exam preparation, homework, lab tests, combining academics with extracurricular activities, and the Viva. Apart from that, most students are stressed because of an "unsystematic study plan," "poor time management," and "overconfidence in the results, etc." These aspects cause frequent stress symptoms such as headaches, fatigue, diarrhea, constipation, anxiety, a disrupted sleep pattern, and a lack of appetite among students [47]. With this regard, Yumba [48] also identified various elements that contribute to academic stress: 1) increasing class load, 2) poorer grades, 3) long hours of study, 4) language challenges, 5) lack of university aid, 6) examination, 7) too many homework assignments, and 8) skipping specific lectures. Unfortunately, this academic stress took a new root because of the pandemic where students were bothered about exam postponement and were concerned about questions such as "How do colleges judge students during admission?" and "Will foreign institutions accept Indian students?" 'When will regular classes resume?' etc. [49]**.** Apart from that many variables that cause test anxiety during the Covid period were also identified which includes "Parents' Pressure," "Covid Fear," "Family Problem" owing to financial concerns, and "No Preparation," and all these aspects enhance academic stress among students [50]**.** Hence, online exam anxiety rose, resulting in low academic performance and increased academic stress [51]. Based on the above discussion H2 has been developed:

H2 Exam anxiety has an impact on academic stress.

c) Exam Anxiety and Coping Strategies:

Previous research has shown that a modest degree of exam anxiety motivates students to accomplish academic achievement, whereas students with a higher level of exam anxiety exhibit unfocused, restless, and squirmy behavior when judging situations [52]. These exam anxieties caused students to become easily distracted throughout exams, fail to grasp relatively basic questions, and had difficulty establishing or retaining relevant knowledge [53]. Moreover, extreme anxiety can prevent judgments, produce a negative frame of mind, and cause poor exam performance [54]**.** As a result, taking proper measures to manage these issues is a critical feature that can be experienced through coping mechanisms. Coping is a fundamental process that describes how one can detect, assess, deal with, and learn from stressful situations. Some of the coping strategies include cognitive therapy, problem-solving, information gathering, emotional evacuation, resistance, detachment, acceptance, seeking social support, and denial. In support of this discovered 79 main coping strategies coalesced into five strategies to alleviate exam anxiety, including "positive thinking, relaxation, preparation, surrender, and attention." also stated that when confronted with a circumstance that they believe will cause them anxiety, the most common response is to avoid the subject and the stress. During the lockdown, students spent most of their time on the internet gaming, and social networking sites rather than on academics; they also felt uncomfortable with online classes [55], and this increased academic stress among undergraduate students. With this regard, the selection of coping strategy was found to be determined by the level of exam anxiety, those who have high test anxiety tend to report much more coping strategies than people who have low test anxiety which the former tended to report using preparation and concentration tactics more frequently as a coping strategy. Previous studies have shown that high exam anxiety leads to high adoption of coping strategies. Hence researchers attempt to analyze this relationship by taking the previous studies into consideration, which gives us a direction to assume the following hypothesis.

H3 The level of exam anxiety has a significant influence on the adoption of coping strategies.

d) Coping Strategies and Academic Stress:

According to Sarros & Densten [56], the "Ways of Coping" include a distinction between two basic forms of coping: problem-focused coping and emotion-focused coping. Problem-focused coping attempts to resolve issues or alter the cause of stress. Emotion-focused coping, on the other hand, seeks to lessen or regulate the emotional suffering connected with the event in general. But in the context of this study Coping Mechanisms or Protective Actions are steps implemented by the government, college administration, and students to lessen Covid anxiety and fear of uncertainty, as well as to reduce high academic stress [57]**.** As Academic stress is a function of mental anguish associated with educational tasks, catastrophes, or even the knowledge of the likelihood of academic failure [58], students typically manage it by looking for something positive in the problem at hand, attempting to cast the stressful circumstance in a distinct light to make it appear more optimistic, drawing some lessons from the stressful experience, and attempting to grow as a person as a result of the experience [59]**.** The implication is that students utilize their actions or thoughts to decrease or remove the emotional impact of anxiety. In addition, it was also argued that it is vital for students to use one’s actions or thoughts to relieve academic stress. Hence it can be postulated that.

H4 The adoption of a coping strategy has an impact on academic stress.

The students and instructors communicating regularly help to alleviate academic stress and allows teachers to track academic success [60]. As per the existing studies, the emotional intensity is increased by the Coping Mechanism, which also provides appropriate counsel for reducing stress [61]**.** Moreover, previous research has focused on how Coping Mechanisms help to reduce stress rather than on how students practice; thus, in addition to assessing the relationship between exam anxiety and academic stress, this study also investigates the role of coping mechanisms in mediating the relationship between study variables. As the review of the literature found the influence of online exam anxiety on coping strategies and influence of coping strategies on academic stress as well as the direct impact of online exam anxiety on academic stress, the researchers aimed to analyze the mediating role of coping strategies in reducing the negative consequences of online exam anxiety on academic stress. Hence it is assumed that.

H5 Stress coping strategies mediate the relationship between exam anxiety and academic stress

From the reviewing literature it come to know that there is some research conducted on the impact of the COVID-19 pandemic on academic stress and coping strategies, there is a need for further exploration of the mediating role of coping strategies in this relationship. Specifically, previous studies have mainly focused on the direct effects of the pandemic on academic stress, without examining the potential role of coping strategies in mitigating or exacerbating these effects. Thus, there is a research gap in understanding the extent to which coping strategies mediate the relationship between the pandemic and academic stress. Additionally, there is a need for research that examines how different coping strategies may have varying effects on academic stress, as well as the potential moderating role of individual differences in coping strategies and stress responses. Understanding these factors can inform the development of effective interventions for promoting resilience and reducing academic stress during the pandemic.

### 1.2 Model

From the evidence of the literature review and the analysis of previous conceptual theories, it came known that exam anxiety has a direct negative impact on academic stress [62]. Further, recent studies [63,64] have added a coping strategy as a mediating variable, and results showed a decrease in academic stress with the mediation of coping strategy. However, these causes and effects are variated among demographic variables being moderate variables. With this assumption, we developed a conceptual model to test the model.

**Fig. 1:**
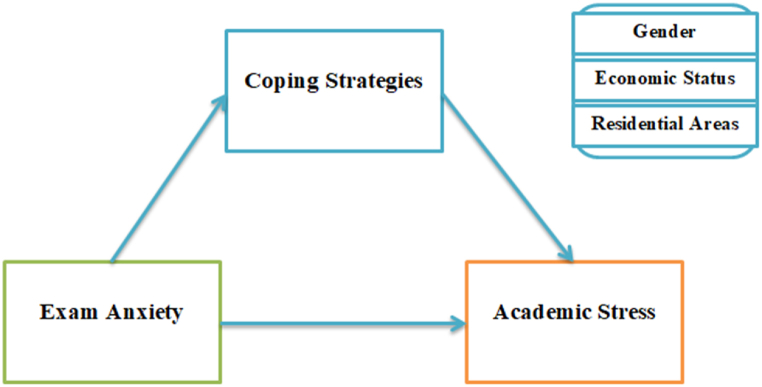
Conceptual model.

Fig. 1 demonstrated the negative relationship between online exam anxiety and academic stress. Furthermore, a high level of exam anxiety causes high adoption of coping strategies indicating a positive relationship. At last, the adoption of coping strategies further has an impact on academic stress. These relationships entailed the researchers assuming a mediating role of coping strategies along with the moderating role of gender, economic status, and residential areas; based on which the above model was developed.

To examine the relationship between the selected constructs, the further organized analysis will be built on the foundation of confirming the validity and reliability of the selected constructs. Based on the many connected works, three constructs-Academic stress (16 items), Online Exam Anxiety (9 items), and Coping Strategy (12 items)-have been identified. When the alpha value is more than 0.70, the study is considered to be reliable (Table 1). The aforementioned test has been employed as a reliability test to evaluate the internal consistency of the data. As observed Cronbach's value for Academic Stress is 0.941, Online Exam Anxiety is 0.932, and Coping Strategy is 0.939. If the Cronbach Alpha value is more than 0.7 then the test result is accepted as they are significant (J. Hair et al., 2017; J. F. Hair et al., 2017). All three Cronbach's values (more than 0.9) are higher than the minimum criteria. The results show that all the aforementioned constructions and indicators satisfy the fundamental requirements because their alpha values are greater than 0.7. Convergent validity was assessed by examining the factor loadings and average variance extracted (AVE) of the constructs. Each parameter strongly loaded onto its associated latent structures, as seen in the aforementioned table, with values ranging from 0.748 to 0.820 (P.001). The AVEs for each component that was more than or equal to 0.50 were academic stress (AVE: 0.820), online exam anxiety (AVE: 0.748), and coping strategy (AVE: 0.812), further demonstrating the constructs' convergent validity. The Maximum Shares Variance and Average Variance Extracted can be compared to assess the discriminant validity, according to Hair et al. (2010). The least Maximum Shared Variance of any item was shared by academic stress (0.771), online exam anxiety (0.736), and coping strategy (0.711), supporting the discriminant validity of the constructs. The measurement model consequently displayed good construct validity and advantageous psychometric properties.

**Table 1:** Reliability and validity test results.

Construct	No. of Items	Reliability		Validity			
		Composite Reliability	Cronbach Alpha	AVE	MSV	Convergent	Discriminant
Academic Stress	16	0.941	0.923	0.820	0.771	AVE>0.5	MSV < AVE
Online Exam Anxiety	9	0.932	0.928	0.748	0.736	AVE>0.5	MSV < AVE
Coping Strategy	12	0.939	0.938	0.812	0.711	AVE>0.5	MSV < AVE

Source: Data Analysis.

## 2 Research methodology

### 2.1 Methods

The current study is descriptive and explanatory in character, and it employs both descriptive and inferential analysis to address the research questions. The research was carried out in Karnataka, a state in southern India with over 5 million students pursuing higher education. Data related to perceived ‘Exam Anxiety’, ‘Academic Stress’, and ‘Coping Strategy’ was collected through a developed questionnaire and later quantified for the purpose of analysis.

### 2.2 Respondents’ profile

The present study considered higher education (Under-graduation and Post-graduation) students of Karnataka, India as participants. Since the researcher did not know the size of the population, the Z score formula (SS = [Z2p (1 p)]/C2) was employed to calculate the sample size, and a reliable size of 431 was determined. Out of 431 students, 60.2% of the respondents are female and most of the respondents come under the age group of 20 and 22. Most of the students (53%) are from the Other Backward Class (OBC) followed by 38.1% and 8.6% of the students from our General and reserved categories respectively. The evaluation of the financial and residential status of the students showed that most of the students (80%) have a moderate financial situation and 47.6% of the respondents are living in rural areas. The educational background of the students showed that 51.8% of the students are pursuing a postgraduate degree and 30.7% of the students are pursuing an under-graduation degree. Most of the respondents (37.1%) are studying in the field of social science and 22.9% of the students are studying in the field of medicine and engineering. The demographic distribution of the students showed sample adequacy in each stratum, which was scientifically evident to test the objectives and hypothesis.

### 2.3 Procedure

The present study followed very systematic and scientific procedures followed to collect the data from the students. Such as.

*First Step:* Before conducting the main study, researchers conducted the pilot study by taking 15 students from Mangalore University Campus (Karnataka, India) to check the content validity and reliability of the questionnaire. Where researchers removed some of the items which were double-barreled, difficult to understand, and irrelevant to the study (From April 2020 to June 2020).

*Second Step:* Entire population is divided into 4 strata (Based on the revenue division of Karnataka) Namely, Bangalore Division, Mysore Division, Belagavi Division, and Kalburgi Division. Later, the researcher collected the population size (Number of Higher education Students) of each stratum through RTI inquiry and decided to distribute 100 questionnaires to each stratum. The sampling unit was selected randomly through convenience sampling techniques (Students who are ready to give opinions voluntarily and satisfy the condition of the study population (From July 2020 to August 2020).

*Third Step:* We were not able to collect data personally (face-to-face) from students because of the third wave of Covid in Karnataka and facing strict lockdown regulations. Therefore, a questionnaire was developed in Google Forms and distributed through WhatsApp, email, and Telegram groups to students. Each author was assigned one stratum (E.g.: Author1 was assigned to Bangalore Division) for contacting students through social media or calls and distributing questionnaires through online platforms (From September 2020 to December 2020).

*Fourth Step:* We kept 381 responses out of 400 received questionnaire issued and in this process responses of missing data, and biased responses (the same questions were asked two times and checked whether they gave the same response and removed if there is a difference in the answer) are deleted for further analysis. The responses in google Forms were downloaded into excel form and coded into numbers (E.g.: Male = 1, Female = 2). Later, these data were imported into SPSS-20 and AMOS graphics for descriptive and inferential analysis (From January 2021 to June 2021).

*Fifth Step:* further imported data was analyzed using various statistical tools such as Two-way MANOVA, Simple Linear Regressions, Structural Equation Model and Multi-Group Comparison. The above statistical tools have been opted by the researcher after checking the normality and multicollinearity assumptions which was within the statistical range, based on which the above parametric tests have been used. Firstly, in order to check the difference in online exam anxiety, Academic stress, and Coping strategy among demographic variables, two-way MANOVA was used which analyses the impact of two independent variables on two or more dependent variables (i.e. online exam anxiety, academic stress and coping strategies. Secondly we intended to examine the direct impact of exam anxiety on students’ academic stress; exam anxiety on coping strategies and the adoption of coping strategies on their academic stress, for which simple linear regression have been used considering each impact as an individual model. Moreover we attempted to analyze the critical mediating role of coping strategy for which structural equation modeling has been run where the model was further tested to check its model fit. Lastly, as literatures showed a significant moderating role of gender, economic status and residential areas of the respondents, the significant existence of its moderating role was unavoidable, for which we conducted multi-group comparison.

## 3 Measurements

### 3.1 Exam Anxiety

Exam Anxiety variable was measured through the items generated by Arora et al., Agius et al., Chhetri et al., and Kumar et al. [49,65,66]. A total of Nine items were taken from the literature to measure ‘Exam Anxiety’ and asked students the five-point Likert scale statements ranging from 1 (Strongly Disagree) to 5 Strongly Agree. The items include both positive and negative statements. Later researcher reverse-coded the negative statements. Exam Anxiety Score was calculated from the response which was ranging from 9 to 45 and later classified into three groups. If the mean score of respondents is between 9 and 21 it was considered as low exam anxiety exists among students, if the mean score is between 21.1 and 33 then moderate exam anxiety exists and if the mean score is 33.1 and 45, it means high exam anxiety exists among students. The format of the Exam Anxiety Scale is depicted in Table 2.

**Table 2:** Exam anxiety scale.

Sl. No	Statements (items)	SA	A	N	DA	SDA
1	I get nervous about the unknown aspect of the online mode of examination					
2	Recently I have fear about being unable to complete the exam					

### 3.2 Coping strategy

The Coping Strategy Scale was built by adapting the items from the study of Awoke et al., Riveiro et al. and Stöber [[67], [68], [69]], in the area of coping strategy during pandemic situations and little modifications in a statement to make a parallel with the covid situation by considering recent studies of Faize & Husain and Joshi et al. [57 ,^70^]. A total of 12 items were used to measure the level of Coping Strategy ranging from 1 (Strongly Disagree) to 5 (Strongly Agree). The items include both positive and negative statements. Later researcher reverse-coded the negative statements. The Coping Strategy Score was calculated from the response which was ranging from 12 to 60 and later classified into three groups. If the mean score of respondents is between 12 and 28 it was considered a low-level coping strategy adopted by students, if the mean score is between 28.1 and 44 then a moderate-level coping strategy is adopted by students and if the mean score is 44.1 and 60, it means a high level of coping strategy adopted by students. The format of the Coping Strategy Scale is depicted in Table 3.

**Table 3:** Coping strategy scale.

Sl. No	Statements (items)	SA	A	N	DA	SDA
1	I am ready to take advice from others to do an action that helps them to reduce stress					
2	I would talk to someone to find out more about the situation					

### 3.3 Academic stress

Academic Stress is considered a construct to understand the stress among student groups during the pandemic where the face-to-face teaching-learning process transformed into online teaching-learning mode. The academic Stress Scale is directly adapted from the study conducted by Zurlo, et al. [71]. This scale consists of 16 items and asked the respondents on a five-point Likert scale ranging from 1 (Strongly Disagree) to 5 (Strongly Agree). The items include both positive and negative statements. Later researcher reverse-coded the negative statements. Academic Stress Score was calculated from the response which was ranging from 16 to 80 and later classified into three groups. If the mean score of respondents is between 16 and 37.33 it was considered as low-level Academic Stress exists among students, if the mean score is between 37.34 and 58.66 then a moderate level of Academic Stress exists among students and if the mean score is 58.67 and 80, it means a high level of Academic Stress exists among students. The format of the Academic Stress Scale is depicted in Table 4.

**Table 4:** Academic stress scale.

Sl. No	Statements (items)	SA	A	N	DA	SDA
1	I am concerned about not being able to meet and speak to their tutors/lecturers personally					
2	Recently, I am getting fear of failing exams					

### 3.4 Sources of data collection

The data for the present study were collected from both primary and secondary sources. Primary data was collected through a well-structured questionnaire, while secondary data was taken from published articles in reputed journals of Scopus and Web of Science databases, periodicals, newspapers, books, and magazines.

### 3.5 Treatment of missing data and common bias

Complete Case Analysis method followed to treat missing data. Researcher collected responses more than sample size. Later eliminated cases with missing data from the analysis, which eliminate potential bias in the results. To eliminate common bias, we followed double blind studies, where neither the participants nor the researchers know which participants are in the control group and which are in the experimental group. This can help to eliminate bias that may arise from the expectations of the participants or researchers.

### 3.6 Ethical consideration

The questionnaire used in this study was approved by the academic integrity and ethical committee of Kingdom University in accordance with the university's research policy and procedures on December 10, 2022. Due to the lockdown in the institution, prospective ethical approval could not be obtained. Therefore, retrospective ethical approval was obtained from the Academic Integrity and Ethics Committee, College of Business Administration, Kingdom University, Bahrain on November 20, 2022 (CBA/17/22). Prior to distributing the questionnaire, the researcher obtained oral consent from the students and informed them of the research's purpose. The respondents gave their consent voluntarily, without any coercion. To protect the privacy and rights of the respondents, all personally identifiable information, such as names and contact details, were kept confidential.

### 3.7 Testing the assumption of normality

**Figure A3:**
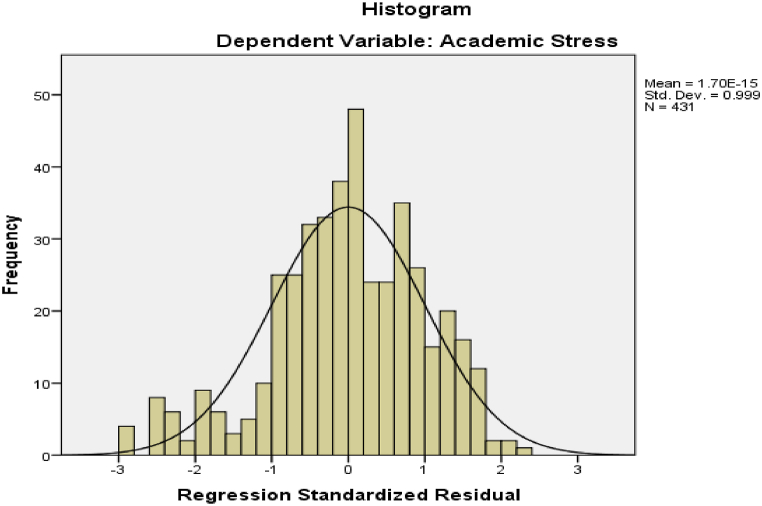
Normal Probability Curve Between Exam Anxiety and Academic Stress.

Kolmogorov-Smirnov (K–S) and Shapiro-Wilk tests were used to ensure the normality of the data. The alternative hypothesis claims that there is a considerable deviation from normalcy. The skewness and Kurtosis values were also considered while determining the data's normalcy. Data is normal if skewness is between −2 and +2 and kurtosis is between −7 and +7 (J. F. Hair, 2020) (Cobanoglu et al., 2019), To fill in the assumption of normalcy, the Kolmogorov-Smirnov (K–S) and Shapiro-Wilk tests are used. The current metric data meets the normal distribution assumption in Figs. A3, A5 & A7 provided in Supplementary materials.

### 3.8 Linearity

**Figure A4:**
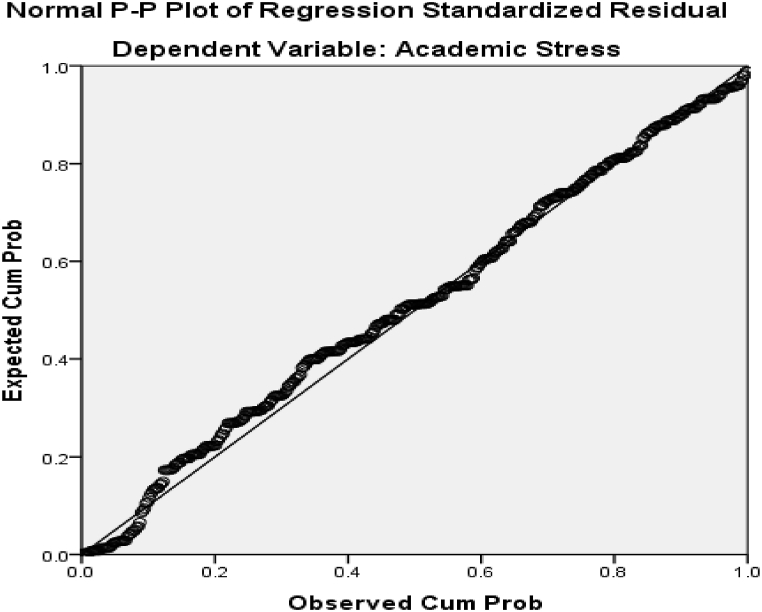
Linear connection Curve Between Exam Anxiety and Academic Stress.

**Figure A5:**
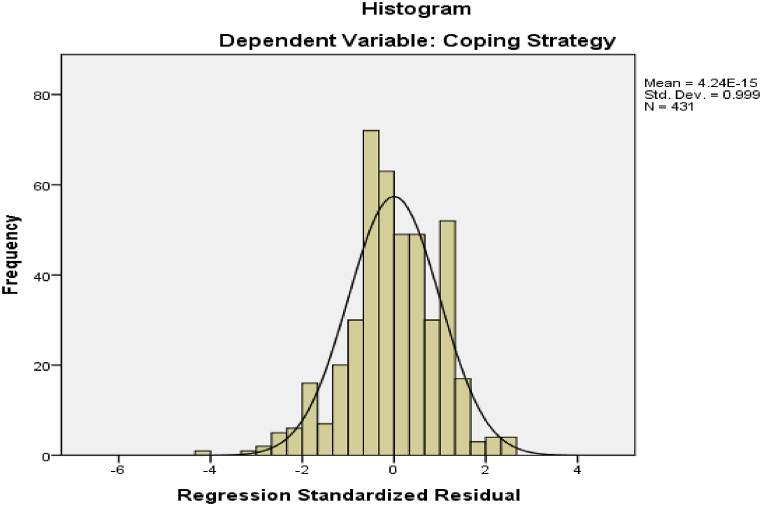
Normal Probability Curve Between Exam Anxiety and Coping Strategy.

**Figure A6:**
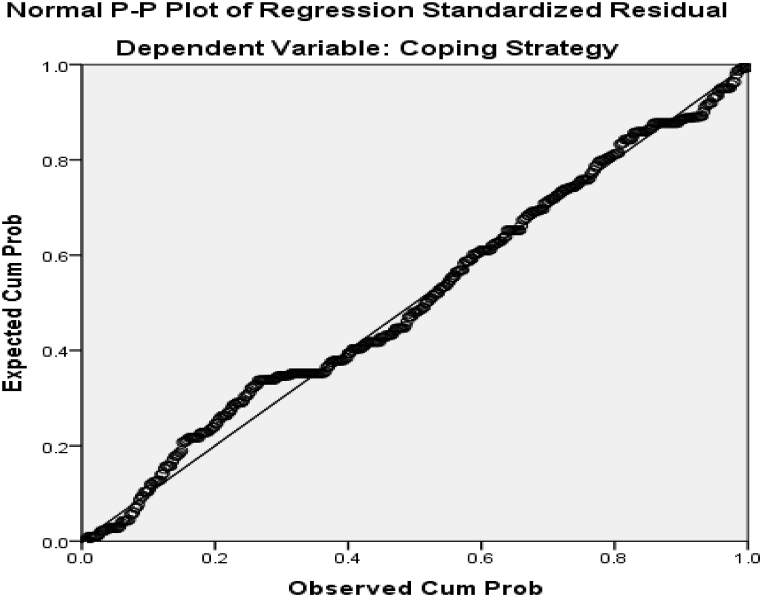
Linear connection Curve Between Exam Anxiety and Coping Strategy.

**Figure A7:**
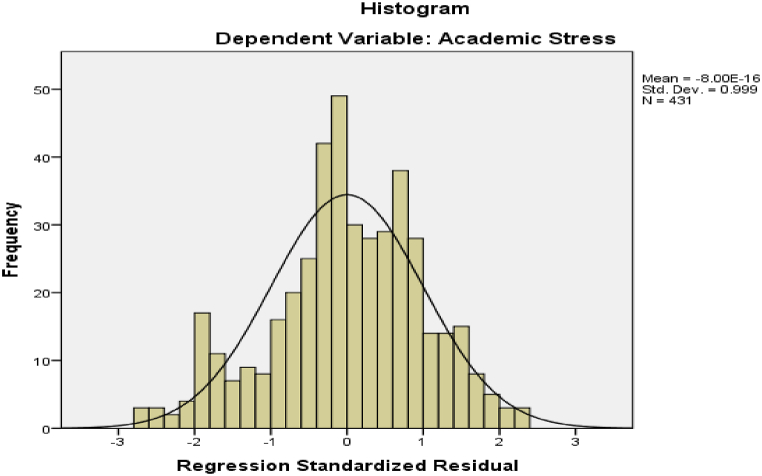
Normal Probability Curve Between Coping Strategy and Academic Stress.

The linearity test is crucial for ensuring the regression analysis assumptions are met by the collected data. There are linear associations that exist between exam anxiety and academic stress, and also between academic stress and coping strategy. Exam anxiety is shown to have a linear connection with coping strategies in Figs. A4, A6, & A8 Supplementary materials. The linearity assumption was met since all of the variables formed a linear relationship.

### 3.9 Histogram for normality

The normal distribution of the data is represented in a histogram with multiple dependent and independent variables. There are data normally distributed between Academic Stress and Exam Anxiety, Academic Stress and Coping Strategy, and Coping Strategy and Exam Anxiety.

## 4 Results

### 4.1 Demographic profile

Demographic details were collected to check the distribution of respondents and to avoid personal bias and concentration of responses. The demographic distribution of the students showed that 60.2% of the respondents are female and most of the respondents come under the age group of 20 and 22. Most of the students (53%) are from the Other Backward Class (OBC) followed by 38.1% and 8.6% of the students from our General and reserved categories respectively. The evaluation of the financial and residential status of the students showed that most of the students (80%) have a moderate financial situation and 47.6% of the respondents are living in rural areas. The educational background of the students showed that 51.8% of the students are pursuing a postgraduate degree and 30.7% of the students are pursuing an under-graduation degree. Most of the respondents (37.1%) are studying in the field of social science and 22.9% of the students are studying in the field of medicine and engineering. The demographic distribution of the students showed sample adequacy in each stratum, and which was scientifically evident to test the objectives and hypothesis.

### 4.2 Learning status and academic sphere during the lockdown

The shutting of schools, universities, and educational organizations across India, and upholding social distancing as a preemptive and precautionary measure against COVID-19, have all transformed the mode of teaching from a conventional standard system to a virtual and online framework. Such a quick and sudden shift in the educational system might cause dramatic changes in the learning methods and scope of teaching. The study observed the changes in the modes of learning, which found that 56.6% of the students of India prefers Online sources rather than hard copy of textbooks. The students also preferred a separate room for study but only 49.9% of the students are able to get separate rooms and other students had to share the room which caused more stress among students. When the researcher asked about tools and techniques used for an online class and their experiences in using such tools, the results exhibited that 84.9% of the students have their own devices, and the rest of the respondents borrowed the device from their friends and relatives. The majority of the students (96.2%) used android mobile phones for online classes and only 3.8% used laptops or Personal Computers. When asked about their prior experience with online classes, only 32% of the students have attended online classes before the outbreak of COVID-19. This made students struggle with the technicality of online classes at the beginning stages. In addition to this, students worried about the completion of the syllabus and the management of study hours. The study found that the majority of the colleges were able to complete only 50–70% of the syllabus and only 18.1% of the college’s completed the full syllabus. The study showed a reduction in study time during the lockdown, which identified that 59.4% of the students studied for fewer hours than in a normal condition (non-Covid setting) and only 15.1% of the students can be able to read for more hours than normal situation. While observing the attendance of the students in an online class, it revealed that 65.9% of the students were able to attend classes daily and 28.1% of the students were able to attend only 3–5 days a week.

The study also tried to identify the challenges and prospects faced while attending online classes. The result showed that 66.4% of the students had issues with internet access and 13.2% of the students were not able to communicate with teachers. While observing the emotional problems faced by students and it revealed that 60.8% of the students opined that they felt stressed, depressed, or anxious during the lockdown. 6.2% of the students opined that the uninterest of professors in taking online classes is also the reason for frustration with online classes. The study tried to identify the most significant stress element in the contemporary academic setting, the study results showed that Study burden (56.9%), Financial pressure (35.4%), and Work-study life balance (30.4%) are the major elements of the stress in an academic setting. As there is saying “reaction for action”, the study identified the students’ social repercussions of stress, which showed that 50.4% of the students had trouble related to listening and 42.2% of the students had trouble sharing ideas. Due to social distance and other covid protocols, 29.5% of the students had trouble withdrawing or isolating from others and 28% of the students had trouble collaborating.

From the above analysis, it can be concluded that the damage of Covid was not only limited to the physical health of the students but also a had negative impact on the learning sphere and feelings of the students.

Descriptive Statistics on Academic Stress, Online Exam Anxiety, and Coping Strategy.

Academic stress, exam anxiety, and coping strategy are measured using a five-point scale ranging from 5 (Strongly Agree) to 1 (Strongly disagree). The descriptive analysis of Exam anxiety showed that students agreed that they get nervous about the unknown aspect of the online mode of examination (3.55 ± 1.294), and they opined that they be afraid about being unable to complete the exam (3.56 ± 1.224), Not able to access the question paper (3.45 ± 1.27), Not able to upload answer script (3.55 ± 1.26) and fairness of online mode of examination. The Overall mean and standard deviation of exam anxiety is 3.49 ± 1.01, which indicates that students have a high level of Exam Anxiety. The descriptive Analysis of Academic stress showed that students agreed about the increase in anxiety level during the exams (3.76 ± 1.26) and they felt inferior when compared to other students while writing exams (3.27 ± 1.25). Students also agreed that they feel very panicky about surprise exams (3.65 ± 1.19), fear failing exams (3.22 ± 1.28), get depressed after taking a test (2.98 ± 1.33), and are concerned about not being able to meet and speak to their tutors/lecturers personally (3.45 ± 1.22). The overall mean and standard deviation of Academic Stress is 3.33 ± 0.84, which indicates that students faced a high level of Academic stress during the lockdown. Further, the descriptive analysis of coping strategy showed that students are ready to take additional action to get rid of the Academic Stress and anxiety problem (3.42 ± 1.15) and they are taking direct action to get around problem (3.45 ± 1.055). The majority of the students are ready to take advice from others to do an action that helps them to reduce stress (3.44 ± 1.16) and they opined that they would talk to someone to find out more about the situation (3.55 ± 1.138). The overall mean and standard deviation of the Coping Strategy is 3.49 ± 0.86, which indicates that students were following a high level of Coping strategy during the Covid pandemic situation.

### 4.3 Hypotheses testing

To support the descriptive analysis and to answer the objectives inferentially few hypotheses were developed and tested. The results and analysis are discussed in the following part.

H1 *There is a significant difference in exam anxiety, Academic stress, and Coping strategy among demographic variables*

Two-way MANOVA was used to test the difference in Academic Stress, Exam Anxiety and Coping Strategy demographic parameters such as Age, Gender, Social status, Economic status, and Residential status of the students*.*

Annexure 1 exhibits the Two-Way MANOVA results to identify the significant difference in Exam Anxiety, Coping Strategy and Academic Stress among demographic profiles and also tried identifying this difference in the intersection of two demographic parameters. The result indicates that there is a significant difference in exam anxiety and Academic stress among age groups (p < 0.05) and the descriptive result showed that students of the age group of 22 and above (M = 3.54 for Exam Anxiety & M = 3.36 for Academic Stress) are having a high level of exam anxiety and academic stress than other age groups but there is no significant difference in adopting coping strategies among different age groups. Further, it showed that there is no significant difference in Exam Anxiety, Academic stress, and Coping Strategy among the Social, Economic, and Residential status of the students (p > 0.05). With the MANOVA test, it is able to identify the difference in dependent variables and it showed differences in Academic stress in the intersection of age group and economic background, economic and residential status, social and economic status, and again in the intersection of Social, economic and residential. It also found differences in exam anxiety in the intersection of age and the residential status of the students. The descriptive result depicted those students of the age group between 18 and 20 and higher economic background (M = 3.740) and students of lower economic status and semi-urban areas (M = 4.013) had a higher level of academic stress. It also identified higher academic stress among students from a higher economic background of SC and ST social groups and it showed a significant difference in exam anxiety among students of the age group between 20 and 22 who are from semi-urban areas. The R square result showed that 36.3% of Academic Stress, 33.4% of Exam Anxiety, and 28.4% of Coping Strategy explained and influenced by demographic variables. Since it is less than 50% the variance is not significant. From the above analysis, it can infer that there is no significant difference in Exam Anxiety, Academic stress, and Coping Strategy among demographic variables except for some of the situations discussed above. Therefore, the researcher fails to reject the null hypothesis at a 5% significance level.

The Durbin-Watson statistic is applied to examine the occurrence of serial correlation between the residuals. According to (Field, 2013), values below 1 or above 3 are the definite root of concern. If the value ranges from 1.5 to 2.5 is relatively normal. The present study shows the value 1.889, 1.852, and 1.999, which forms an acceptable range between 1.5 and 2.5.

### 4.4 Impact of exam anxiety on academic stress

Table 5 showed a correlation value between exam anxiety and Academic stress is 0.41 (p > 0.01) which showed a positive relationship between variables. The regression coefficient between Exam Anxiety and Academic Stress is 0.410, which reveals that exam anxiety has a significant impact on academic stress. The R^2^ value indicates exam anxiety contributes to Academic Stress by 16.8% and the remaining 83.2% are to other unobserved variables. The ANOVA results showed that the F value is 86.652 and the p-value is less than 0.01, which indicates the model is significant. This means that exam Anxiety (IV) is a highly statistically significant impact on Academic Stress (DV) Since the p-value is less than 0.01, the working hypothesis (H2) is accepted.

**Table 5:** Direct relationship.

Hypotheses	Path	Standardized Estimate	P value	R^2^
H2	Exam Anxiety → Academic Stress	0.410	.000	0.1681
H3	Exam Anxiety → Coping Strategy	0.487	.000	0.237
H4	Coping Strategy →Academic Stress	−0.339	.000	0.115

### 4.5 Impact of Exam Anxiety and Coping Strategy

Table No.5 showed a correlation value between Exam Anxiety and Coping Strategy is 0.487 (p > 0.01) which showed a positive relationship between variables. The regression coefficient between Exam Anxiety and Coping Strategy is 0.487, which reveals that Coping Strategy has a significant impact on academic stress. The R^2^ value indicates exam anxiety contributes to Coping Strategy by 23.7% and the remaining 76.3% are by other unobserved variables. The ANOVA results showed that the F value is 133.443 and the p-value is less than 0.01, which indicates the model is significant. This means that Online exam anxiety (IV) is a highly statistically significant impact on Coping Strategy (DV). Since the p-value is less than 0.01, the working hypothesis (H3) is accepted.

### 4.6 Impact of coping strategy on academic stress

Table 5 showed a correlation value between coping strategy and Academic stress is −0.339 (p > 0.01) which showed a negative relationship between variables. The regression coefficient between Academic Stress and Coping Strategy is −0.339, which reveals that Coping Strategy has a significant impact on academic stress. The R^2^ value indicates coping strategy contributes to academic stress by 11.5% and the remaining 88.5% are by other unobserved variables. The ANOVA results showed that the F value is 55.592 and the p-value is less than 0.01, which indicates the model is significant. This means the Coping Strategy (IV) is a highly statistically significant impact on Academic Stress (DV). Since the p-value is less than 0.01, the working hypothesis (H4) is accepted.

Further hypotheses were developed to identify whether there is a mediation effect between study variables. The testing of the mediation effect is to know the indirect effect of the independent variable on the dependent variable [72,73].

### 4.7 Mediation analysis (hypothesis H5)

**Fig. 2:**
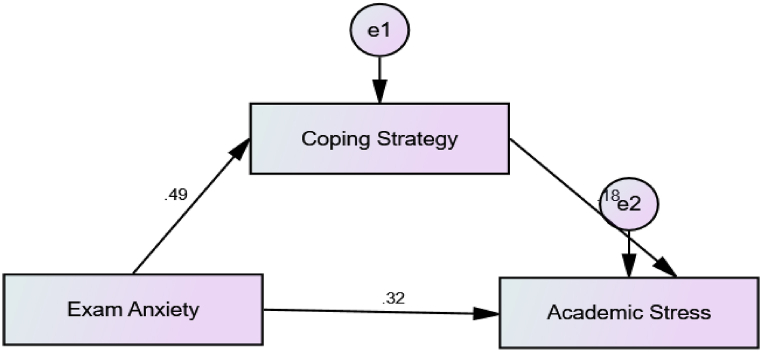
Structural equation model. Source: Data analysis.

The mediating impact was investigated using AMOS 26 and the measurement model was estimated using SEM. The measuring paradigm for Academic Stress, Exam Anxiety, and Coping Strategy is shown in Fig. 2. Academic Stress is measured with sixteen variables, coping strategies with twelve variables, exam Anxiety with nine variables, and the mediation effect result is depicted in Table 6.

**Table 6:** Mediation result.

Effect	Standardized Estimation (SE)	P-value	Results
Effect (Total)	0.41	0.002**	Statistically significant
Effect (Direct)	0.277	0.004**	Statistically significant
Effect (Indirect)	0.133	0.009**	Statistically significant

Source: Data Analysis.

From the analysis of the mediating effect, the study found that exam anxiety has a significant indirect effect on academic stress through the mediation of the coping strategy. Hence, coping strategy plays a significant mediating role between exam anxiety and academic stress (Estimate = 0.133, P = 0.009). Therefore, it can be concluded that from the mediation of the coping strategy the impact of exam anxiety on academic stress is decreased. Hence, H5 is accepted at a 1% significance level.

## 5 Discussion

While the world crumbled around COVID-19, students’ academic lives were severely impacted and disregarded. Students have faced numerous issues as a result of the pandemic, including the conversion of offsite classes to online classes, exam postponement, online seminars and assignments, and online exams. According to Arora et al. [65], COVID-19 has increased exam anxiety among students, and this paper extends the study to assess the impact of exam anxiety on academic stress. This study was initiated to examine how coping strategies can help students cope with academic stress and whether their demography details have any remarkable influence on their exam anxiety, academic stress and coping strategies in this pandemic. The results showed that exam anxiety has a direct impact on academic stress, which supported the study result of Borsheim et al., Karasmanaki & Tsantopoulos, Malhotra, Schleicher and Suresh Prabu [[74], [75], [76], [77], [78]]. As a contradiction to the findings of Malhotra, Yumba, Zeidner, and Zurlo et al. [48,71,76,79], the study revealed a significant difference in exam anxiety and stress among different demographic variables. It was found that students from low-income families and those pursuing post-graduate studies are more stressed. To emphasize the relevance of coping strategies in stress reduction, the study examined the use of coping mechanisms prior to attending a test in accordance with the study of coping mechanisms [80]. Even while the descriptive study found the same conclusion, the inferential analysis found significant discrepancies, notably in the direct influence on academic stress. The study's findings aid educational institutions in better understanding test anxiety among students in COVID-19, as well as estimating the influence of exam anxiety on academic stress. A working model was developed by referring to the study result of Yang et al. and Faize & Husain [70,81], and it revealed that academic stress has a significant indirect effect on online exam anxiety through the mediation of coping strategy.

Furthermore, throughout the lockdown, this study highlighted the learning state and academic sphere. According to studies by Kulal & Nayak, Jervis & Brown, and Karasmanaki & Tsantopoulos [75,82,83], online learning increased because of the pandemic crisis. The study focused on the covering of the syllabus during the lockdown, and it can be concluded that teachers are having difficulty finishing the syllabus, which has undoubtedly raised students' test anxiety and tension. This study added more evidence to the study conducted by Aiyer et al. [44] that during online exams and viva, academic integrity is constantly jeopardized. According to research done by Agius et al. and Arora et al. [65,84], technological limitations are the most significant impediment to the success of online classes, as demonstrated in this study. When looking at the study's overall findings, it's clear that COVID-19 had a bad influence on both mental health and academic life. Even though this study found that coping skills might assist students to cope with test stress, the role of parents, colleagues, and students themselves is unavoidable.

### 5.1 Limitation

The undertaken study made an effort to measure the effect of academic stress on exam anxiety by including a coping strategy. There are several limitations that can influence the interpretation of the present study. However, due to time constraints, for the study, only students of higher education were considered even though the same amount of academic stress and exam anxiety was reported among other students also. That class of students is also exposed to the same teaching-learning environment that of higher education students during the pandemic. Since pandemic largely affected to India and because of cross border travelling difficulty the study is confined to the geographical area being Karnataka state, India. Hence, the study result cannot be generalized to the entire world or similar studies to be taken up in the future as the settings and situation where study will be undertaken may not remain the same.

Theoretical limitations of this study include the use of self-reported data, which may be subject to bias and may not accurately reflect the actual experiences of students. Additionally, the study only examined the mediating role of coping strategies, and did not explore other potential factors that could contribute to perceived academic stress during the pandemic.

Methodological limitations include the cross-sectional design of the study, which only provides a snapshot of the participants' experiences at a single point in time. Longitudinal studies would be needed to better understand the changes in academic stress and coping strategies over time. Additionally, the study relied on a convenience sample of students from a single university, which may limit the generalizability of the findings to other student populations. Finally, the study did not assess the effectiveness of specific coping strategies in reducing academic stress, which could be explored in future research.

## 6 Conclusion

In conclusion, the present study investigated the impact of exam anxiety on academic stress during the COVID-19 pandemic, and examined the role of coping strategies in reducing stress. The study revealed that exam anxiety has a direct impact on academic stress, and there were significant differences in exam anxiety and stress among different demographic variables, particularly for students from low-income families and those pursuing post-graduate studies. Additionally, coping strategies were found to have a significant indirect effect on online exam anxiety through the mediation of academic stress. The study contributes to the understanding of the impact of the pandemic on students' academic lives and mental health, and provides insights for educational institutions to better support students in coping with test anxiety.

Furthermore, the study highlighted the challenges of online learning during the pandemic, including difficulties in covering the syllabus, jeopardized academic integrity, and technological limitations. These findings add to the existing literature and emphasize the need for further research on the long-term effects of the pandemic on education and mental health.

Overall, the originality of this paper lies in its examination of the relationship between exam anxiety, academic stress, and coping strategies during the COVID-19 pandemic. The study's methodology, including the use of a questionnaire and inferential analysis, was appropriate for the research question and contributed to the novelty of the findings. The study's results provide insights for educational institutions and policymakers to better support students during and after the pandemic.

### 6.1 Practical implication

This study has significant theoretical, practical, and social implications. From a theoretical perspective, the findings of this research contribute to the growing body of knowledge about academic stress among higher education students during COVID-19. The study's results will be useful for academicians, practitioners, and policymakers who aim to improve higher education over time. During a pandemic outbreak, higher education institutions play a critical role in reinforcing their blended learning strategies. Therefore, educational institutions must develop innovative and comprehensive approaches to promote and treat mental health concerns among students. As online education is still in its infancy, educational institutions should train students and teachers in this area during the paradigm shift from offline to online blended learning content delivery mode. The abrupt transition in the form of education significantly impacts college students' mental health, and they require support from both their family and college. Therefore, any new virtual learning platform or e-learning policy change should consider students' psychosocial circumstances. The government and educational institutions should collaborate to provide high-quality emergency psychological assistance to students when needed.

Institutions of higher learning could practically target academic stress and promote mental health among students by developing and implementing evidence-based programs, such as stress management and coping skills training, mindfulness-based interventions, and psychoeducational workshops focused on mental health promotion. Moreover, institutions could establish support groups or peer mentoring programs to provide additional support for students. For clinical recommendations, teletherapy or online counseling services could be implemented to provide accessible and convenient mental health support for students. Mental health professionals could also collaborate with academic advisors and faculty to provide early intervention for students who may be struggling with academic stress and related mental health concerns. Further research could explore the long-term effects of the COVID-19 pandemic on higher education students' mental health and the effectiveness of different intervention strategies for addressing academic stress among students during the pandemic. Management recommendations could include the development of policies and procedures that prioritize student mental health and well-being, such as the establishment of mental health task forces, the allocation of resources for mental health support, and the inclusion of mental health services in institutional budgets.

Looking ahead, the future scope of this study could include examining the long-term effects of the COVID-19 pandemic on the mental health of higher education students. Additionally, future research could explore the effectiveness of different intervention strategies for addressing academic stress and related mental health concerns among students during and after the pandemic.

## Author contribution statement

Habeeb Ur Rahiman: Conceived and designed the experiments; Performed the experiments; Analyzed and interpreted the data; Contributed reagents, materials, analysis tools or data; Wrote the paper.

Niyaz Panakaje: Conceived and designed the experiments; Performed the experiments; Analyzed and interpreted the data; Wrote the paper.

Abhinandan Kulal: Conceived and designed the experiments; Analyzed and interpreted the data; Wrote the paper.

Harinakshi Suvarna: Performed the experiments; Contributed reagents, materials, analysis tools or data; Wrote the paper.

S M Riha Parvin: Performed the experiments; Analyzed and interpreted the data; Contributed reagents, materials, analysis tools or data; Wrote the paper.

## Declaration of competing interest

The authors declare that they have no known competing financial interests or personal relationships that could have appeared to influence the work reported in this paper.

## Figures and Tables

**Figure A8:**
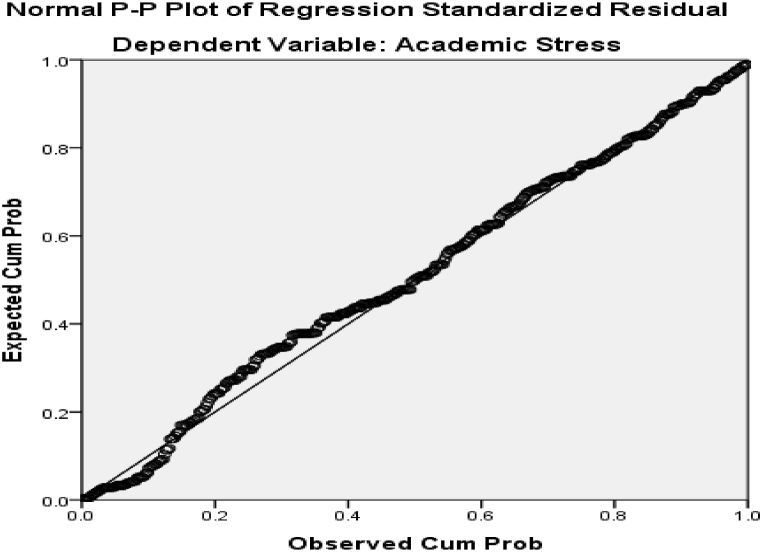
Linear connection Curve Between Coping Strategy and Academic Stress.
